# Self-Perception of Parental Role, Family Functioning, and Familistic Beliefs in Italian Parents: Early Evidence

**DOI:** 10.3389/fpsyg.2015.01983

**Published:** 2016-01-11

**Authors:** Elisa Delvecchio, Daniela Di Riso, Silvia Salcuni

**Affiliations:** Department of Developmental Psychology and Socialization, University of PaduaPadua, Italy

**Keywords:** parenting, family adjustment, cultural values, familism, Italian parents

## Abstract

Recent research has explored the relationships between family and cultural issues, claiming attention on the need to consider and evaluate cultural values and beliefs as useful factors to promote positive family adjustment and parenting outcomes (Cardoso and Thompson, 2010; Taylor et al., 2012). This paper explored self-perception of parental role, family maladjustment and cultural beliefs in a sample of Italian parents. More specifically, 204 mother and 204 fathers of adolescents (13–17 years old) filled self-report questionnaires about family system maladjustment (Family Assessment Measure-III), self-perception of parental role (Self-Perception of Parental Role), parents’ beliefs and attitudes toward the family (Attitudinal Familism Scale), and parents’ cultural values (Cultural Values Survey). Results showed that parents have a similar self-perception of family functioning and they share common cultural beliefs and values toward the family. However, fathers felt more satisfied and involved in parenting then mothers and they were more able to balance the different roles of their life. Mothers and fathers showed a similar path of correlations, in which greater level of satisfaction in parenting and better ability in role balancing correlated with a more positive family adjustment. Moreover, a higher perception of family maladjustment was associated to lower levels of family cohesion and cooperation. Furthermore, higher levels of satisfaction were associated to higher scores in family solidarity, equality among sexes and equality in decision takers. These results introduce important implications for family studies in Italian culture, and open to comparison with parenting in other cultures.

## Introduction

The last two decades have been marked by a substantial attention to children’s well-being that can be generally understood to encompass physical, social, and emotional health (Statham and Chase, 2010). Several authors suggest that family functioning and parenting practices have an enormous influence over the development, education, health, and well-being of their children (Brown, 2004; Amato, 2005; Armstrong et al., 2005; Nelson et al., 2014). More specifically, plenty of studies demonstrated that negative family functioning and unhealthy parenting are often linked to worse emotional and behavioral outcomes for a child, including more opportunities for conduct problems, peer problems, eating disorders, substance abuse, internalized problems (i.e., anxiety and depression), and less positive outcomes such as diminished social competence and self-esteem later in life (Scaramella et al., 1999; Smetana et al., 2002; Barnes et al., 2006; Dishion et al., 2008; Abu-Rayya and Yang, 2012; Letourneau et al., 2013; Ferro and Boyle, 2014; Angley et al., 2015). Those findings claim for research in this area, particularly in Italy which was rated in the middle/bottom third of the rank in a recent report on well-being in European children (Bradshaw and Richardson, 2009).

Family functioning is defined as the interactions between -and reactions to- family members; it includes variables within the family such as members’ roles and boundaries, degree of cohesion, adaptation, and resilience, as well as family values and beliefs (Winek, 2010). In other words, as stated in the final report about positive family functioning edited by the Australian Department of Families, Housing, Community Services and Indigenous Affairs (2010), family functions refers to a variety of characteristics encompassing several domains such as emotional attributes (e.g., closeness of parent–child relationships, warmth, sensitivity, perceived support, and safety), family governance issues (e.g., members’ role, age appropriate rules), engagement and cognitive development, physical health habits, quality of intra-familial relationships (e.g., parent–child interactions, parent-parent relationships, spouse–spouse relationships), and social connectedness (e.g., relationships with the extended family, activities outside the family unit, members’ role balance).

A good family adjustment provides a foundation for positive parenting (Newland, 2015). Parenting is a multidimensional challenging process that covers complex variables, not limited to caregiving activities (Bornstein, 2002). It is often assessed focusing on parents’ perception of their ability to perform as parents (Waldman-Levi et al., 2015). Most of the research on self-perception of parenting were based on self-efficacy theory, stating that individuals who perceive themselves as competent and valuable, who are self-confident with their abilities, and who are able to envisage the likely effects of their actions, will, as parents, more probably act as positive and helpful partners for their children’s development (Bandura, 1997; Bornstein et al., 2011b). Self-perception of parenting includes several possible characteristics such as feeling of competence experienced in the role of parent, involvement in caregiving, satisfaction from caregiving relationship, and ability to balance parenting with other role in life (Bornstein et al., 2003).

Recently, researchers have begun to claim attention on the need to consider and evaluate cultural values and beliefs as useful factors to promote positive family and parenting outcomes (Cardoso and Thompson, 2010; Taylor et al., 2012). Bornstein et al. (2011b) well motivated this issue, highlighting that “adults do not parent in isolation, but always do so in social and cultural context.” Thus, parents and cultures are strictly related since parenting, among other things, is aimed to transmit the prevailing culture across generations and to transmit the existing culture to the next generation. Culture is here contextualized as a set of distinctive patterns of beliefs and behaviors that are shared by a group of people and that serve to regulate their daily living, including practices related to childrearing and child development (Bornstein et al., 2011a). An important cultural value that has been found positively related to family adjustment is familism, a cultural belief originally devised for Latino populations, describing the strong identification of individuals with their family (Sabogal et al., 1987). Familism emphasizes an ideal for family relationships to be warm, close, and supportive and that is considered one of the several family-related constructs that are prevalent in collectivist cultures that value prioritizing family over self (e.g., Lugo Steidel and Contreras, 2003; Campos et al., 2008; Abdou et al., 2010). Past research reported that familism is linked to positive familial relationships, high family cohesion and harmony (Roosa et al., 2005; Germán et al., 2009; Cardoso and Thompson, 2010). Moreover, familistic beliefs have been found to have a positive association with involvement in parenting (Coltrane et al., 2004; Romero and Ruiz, 2007; Taylor et al., 2012).

Furthermore, although theorists claimed the need to consider both fathers and mothers in research, there is a paucity of papers assessing fathers’ beliefs, behaviors, and attitudes (Marsiglio et al., 2000; Campos, 2008) comparing to the ones devoted to mothers’. Although some studies have demonstrated similar parenting characteristics between mother and fathers (Pleck and Hofferth, 2008), others underlined the specific role of fathers for adolescents (e.g., separation–individuation process) (Pleck, 2007; McBride et al., 2009). For these reasons, we addressed this limitation by examining both parents. Thus, the current study was firstly aimed to compare genuine Italians mothers and fathers focusing mainly on their self-perception of parental role, such as family adjustment and cultural beliefs (e.g., identification of individuals with their family members, family solidarity), instead of on parenting beliefs and practices (Bornstein et al., 2001; Hsu and Lavelli, 2005; Vieno et al., 2009). Although a limited number of authors have already explored familistic values and family functions (Kumpfer et al., 2002; Germán et al., 2009; Taylor et al., 2012), none of them referred to Italian samples. It was hypothesized that mothers and fathers report small differences in cultural beliefs as well as perceiving family functioning (Ferrari, 2002; Svetina et al., 2011; Delvecchio et al., 2014; Laghezza et al., 2014). Moreover, we hypothesized fathers demonstrating to score higher on self-perception of parental role (Pleck, 2007; McBride et al., 2009).

A second purpose was to evaluate the possible link between family functioning, parenting self-perception, and cultural values in mothers and fathers, respectively. Significant positive correlations were expected between family functioning and self-perception of parental role in both parents (Bornstein et al., 2011b; Newland, 2015). Furthermore, parents’ familistic values were expected to be associated with parenting satisfaction and family positive functioning (Cardoso and Thompson, 2010; Taylor et al., 2012).

## Materials and Methods

### Participants

Participants were 408 mother–father dyads (50% mothers).^1^ They were heterosexual couples of adolescents (*M*_age_ = 16.61, *SD* = 1.91, 41% males). Mothers’ mean age was 47.46 (*SD* = 4.22) and fathers’ 49.91 (*SD* = 4.62). All subjects were Caucasian and lived in urban and suburban areas of North and Central Italy. Parents’ socio-economic level, measured by SES (Hollingshead, 1975), was middle to upper for 89% of families; 9% had a low socio-economic status and only 2% reported a very high level. Families were recruited through their children’s schools, and met the following criteria: (a) both mothers and fathers agreed to participate, (b) all were regularly married couples, (c) all participants completed the entire assessment phase, (d) parents and children did not meet criteria for psychiatric diagnosis and were not under psychological treatment. Approximately 84% of the families who received the leaflet agreed to participate. Those who declined adduced reasons such as lack of interest and concerns about sharing personal information.

### Procedures

This study was conducted in compliance with the ethical standards for research outlined in the Ethical Principles of Psychologists and Code of Conduct (American Psychological Association, 2010). Approval by the Ethical Local Committee for Psychological Research was obtained from Padua University (Prot. N. 1523, 2014). Participation in the study was solicited via leaflets. Parents written signed informed consents to participate in the study were obtained before data collection. They completed the questionnaires at home and returned them to the research team through their children. No incentives were awarded and voluntary participation was emphasized. Confidentiality was assured by replacing personal’s information with a numeric code. A forward- and back-translation procedure was used to ensure the linguistic, conceptual, and cultural equivalence of the measures not yet validated in Italian (Peña, 2007; Erkut, 2010).

### Measures

The *Self-Perception of Parental Role* (SPPR; MacPhee et al., 1986) is a 16 items tool developed to assess parental self-perception through four subscales: Investment (five items), Competence (six items), Satisfaction in parenting (five items), and Role Balance (six items). Investment refers to parent’s involvement and commitment to children; Competence assesses parent’s perception of his/her competence in child rearing; Satisfaction investigates how much a parent is satisfied in his/her role; Role balance assesses the way in which a parent balances the possible different roles of his/her life (e.g., parent, spouse, employee, …; Perry-Jenkins et al., 2000). In order to minimize the impact of social desirability, each item has a pair of statements with contrasting endpoints of the dimension in question. As example, one item for satisfaction states “Being a parent is a satisfying experience to some adults BUT for other parents, being a parent is not all that satisfying.” The respondent chooses the statement that describes him/her best, and rated it on a four response items, weighted 1, 2, 4, and 5. The absence of a response indicating that the item was equally like and unlike the respondent was done in purpose. SPPR scales showed good internal reliability, construct validity, and test–retest reliability (MacPhee et al., 1986; Seybold et al., 1991). The Italian translation of the questionnaire was carried out following the guidelines suggested by Van de Vijver and Hambleton (1996). The Italian version was pilot-tested with 36 mothers and 36 fathers; no specific problems emerged. In the current sample, Cronbach’s alphas ranged from 0.59 (competence) to 0.62 (role balance) for mothers, and from 0.52 (satisfaction) to 0.64 (role balance) for fathers.

The *Family Assessment Measure-III General Scale* (FAM-III; Skinner et al., 1983) is a 50-item self-report measure devoted to assess family maladjustment. Participants were asked to answer on a 4-point Likert scale ranging from 0 (completely disagree) to 3 (strongly agree). An example of item is “Family duties are fairly shared.” The current study took into account only FAM-III Total score, which assesses family system shared values, norms, and goals. Higher scores indicate worse family functioning. FAM-III showed good psychometric properties (Skinner et al., 2000; Laghezza et al., 2014). In our study, Cronbach’s alpha for mothers was 0.77 and 0.72 for fathers.

The *Attitudinal Familism Scale* (AFS; Lugo Steidel and Contreras, 2003) is a 18-item scale aimed at assessing beliefs and attitudes toward the family (Schwartz, 2007): Familial support indicated the idea that family members should engage in reciprocity to support each other; Interconnectedness is related to the belief that one should maintain a strong bond with family members; Honor subscale measures the belief that one must maintain family honor; Subjugation subscale measures the idea that one should sacrifice one’s own needs for the family. The scale was developed specifically to capture the Latinos approach to familism. However, the Lugo Steidel and Contreras (2003) scale has been found to have an equivalent factor structure and associations to psychological well-being and distress in Latino, European, and Asian background samples (Schwartz et al., 2010). Participants are asked to answer on a 5-point Likert scale, ranging from strongly disagree (1) to strongly agree (5). A sample item of AFS is “Parents and grandparents should be treated with great respect regardless of their differences in views.” Higher scores denote major levels of familism. AFS showed adequate reliability and validity (Lugo Steidel and Contreras, 2003; Schwartz, 2007). The Italian translation of the questionnaire was carried out following the guidelines suggested by Van de Vijver and Hambleton (1996). The Italian version was pilot-tested with 36 mothers and 36 fathers; no specific problems emerged. In the current sample AFS showed good internal consistency for mothers (α = 0.79) and fathers (α = 0.80).

The *Cultural Values Survey* (CVS; Chia et al., 1994) is a 45-items questionnaire assessing how important is each proposed issue regarding seven areas linked to family: Family Solidarity (eight items), Executive Male Attitude (eight), Conscience (eight items), Equality of Sexes (seven), Temporal Farsightedness (four items), Independence (six items), Spousal Employment (three items). Participants are asked to answer on a 5-point Likert scale ranging from 5 (extremely important/strongly agree) to 1 (not at all important/strongly disagree). Family solidarity indicates the sense of perceived cohesion and coherence in the family system; Executive Male reflects the attitude that men are decision makers and women are homemakers; high scores on this component express agreement for instance with the following beliefs: only girls and woman should do homework; only men could make the most important decision in the family, marriage and children are more important for a girl than a career. Conscience stresses the importance of the family to conform to traditional customs, moral and social standards, to perpetuate the ancestral line, being respected by the community, to respect for the authority. Temporal farsightedness measures the interest in the future compared with the past, and the willingness to delay gratification and finding a place for the family in the community and in relation to the past and the future. Independence indicates how much important are behaviors related to seek fun and excitement, shame from dependence on public welfare a secure and comfortable life, freedom (from parental and similar constraint) and the value of hard work. Finally, Spouse Employment scale measures the tendency to give importance to the opinion that each spouse should decide about her/his own job, not about her/his spouse job. An example of item of this measure is “How important is (…) a cohesive family?” Chia et al. (1994) reported adequate psychometric characteristics for CVS. The Italian translation of the questionnaire was carried out following the guidelines suggested by Van de Vijver and Hambleton (1996). The Italian version was pilot-tested with 36 mothers and 36 fathers; no specific problems emerged. In the current sample, CVS subscales showed adequate internal consistency ranging from 0.53 (independence) to 0.78 (equality of the sexes) for mothers and from 0.56 (temporal farsightedness) to 0.79 (family solidarity) for fathers.

### Data Analysis

The Statistical Package for Social Sciences (SPSS.21) was used to compute descriptive statistics, correlations and paired *t*-tests to compare mothers and fathers medium scores on the selected scales and sub-scales. *T*-test was also used to assess possible gender influence between mothers and fathers’ answers. When *t*-test was significant, effect size was calculated and classified according to Cohen’s (1988) *d* criteria: small effect size, if the *t* ranged from 0.10 to 0.30; medium effect size if *t* ranged between 0.31 and 0.50; large effect size if *t* was higher than 0.50 and very large effect size if *t* was higher than 0.80. Correlations were calculated between self-perception and family perception scores and cultural values variables separately for mothers and fathers. Correlation effect size was classified according to Cohen (1988).

## Results

**Table 1** shows descriptive statistics of all selected measures for mothers and fathers, respectively, and reports the results of paired *t*-tests used to compare their medium scores. Cohen’s *d* value is reported only for statistically significant *t*-test. Furthermore, **Table 1** reported Person’s *r* about correlations of each variable between parents: according to Cohen’s suggestions their effect sizes were medium to high, suggesting a good agreement within the couples, along all variables.

**Table 1:** Means, Standard Deviations, and Paired *t*-test for mothers and fathers for all the selected variables.

	Mothers (*n* = 204)		Fathers (*n* = 204)					
	*M*	*SD*	*M*	*SD*	*t*_(203)_	sign	Cohen’s *d*	Pearson’s *r*
**SPPR**								
Investment	2.78	0.54	3.01	0.70	-3.74	<0.001	-0.37ˆ*	0.12
Competence	3.11	0.71	3.70	0.84	-9.33	<0.001	-0.76ˆ**	0.36ˆ*
Role Balance	3.56	0.72	4.06	0.89	-7.68	<0.001	-0.62ˆ**	0.38ˆ*
Satisfaction	3.99	0.77	4.27	0.77	-4.85	<0.001	-0.36ˆ*	0.43ˆ*
**FAM-III**								
Total score	39.18	10.97	39.39	10.97	-0.26	0.79		0.44ˆ*
**AFS**								
Total score	68.44	8.94	68.84	9.34	-0.63	0.52		0.52ˆ**
Support	18.71	3.27	18.76	3.19	–16	0.87		0.51ˆ**
Interconnectedness	17.54	2.41	16.91	2.79	3.02	0.003	0.23	0.36ˆ*
**Honor**	9.69	2.44	9.85	2.23	-0.93	0.35		0.31ˆ*
Subjugation	7.57	2.13	8.23	2.10	3.56	<0.001	-0.31ˆ*	0.23
**CVS**								
Family solidarity	4.20	0.53	4.12	0.58	2.10	0.04	0.14	0.55ˆ**
Executive male attitude	2.13	0.58	2.33	0.61	-4.54	<0.001	-0.34ˆ*	0.42ˆ*
Conscience	3.46	0.58	3.34	0.67	2.40	0.02	0.19	0.34ˆ*
Equality of sexes	4.29	0.60	4.13	0.69	3.66	<0.001	0.25	0.53ˆ**
Independence	3.27	0.55	3.27	0.54	-0.10	0.92		0.36ˆ*
Temporal farsightedness	3.31	0.61	3.28	0.69	0.61	0.54		0.28
Spousal Employment	2.48	0.73	2.59	0.77	-1.56	0.12		0.20

*Effect size: smallbetween 0.10 and 0.30; ^∗^medium between 0.31 and 0.50; ^∗∗^large higher than 0.50*.

In self-perception of parental role, both parents shared the same level of satisfaction, however, fathers supported that being a parent allows them to be more involved in different roles beside parenthood than mothers. Concerning parenting instead, both parents supported that satisfaction is a more important issue than role balance. Results show significant differences between fathers and mothers regarding the self-perception of parental role (see **Table 1**). Fathers felt more involved, competent and satisfied in parenting than mothers. Furthermore, they reported to be more able in balancing the different roles they take in their life.

Both parents share the same level of attachment to their family members (both nuclear and extended) and a similar sense of identification with those family members (Familism). Looking at the dimensions of the AFS, they share a high similar level of familism account for intense feelings of interconnectedness, that is parents underscored that family members must keep in close emotional relationship and physical contact with other family members. They also share a strong assumption of an obligation to support individual members and give them assistance. However, familism for them does not mean so much maintaining the family honor by behaving in ways that will be looked favorably by other members and/or outsiders (Lugo Steidel and Contreras, 2003), nor they belief that a person must be submissive and yield to the family willingly subordinating individual preferences for the benefit of family.

In respect with cultural values, both parents underscored above all the importance of equality of sex and family solidarity as compared with the other values assessed. They both stressed an agreement with statements that seem to minimize sexual stereotyping (e.g., married women have the right to continue their education, raise children is important for mothers and fathers, it is ok for a married woman with young children to have a job outside the home). They also both stressed the importance of a cohesive, cooperative and harmonious family, respect for elders, education and achievement. Fathers and mothers underscored Executive Male much more than the other values assessed. Instead, both parents did not give so much importance to Temporal Farsightedness, Independence, and Spouse Employment as compared with the other assessed values. Also independence was not too much stressed. Finally, they agreed not to give importance to the opinion that each spouse should decide about her/his own job, not about her/his spouse job. Additionally, four subscales of CVS showed significant results. However, with the exception of the Executive Male Attitude, Cohen’s *d* effect sizes were small, suggesting trivial effects, mainly due to the large sample size. Thus, the only one interpretable result suggests that even mothers and fathers resulted quite similar in executive male attitude, and this is the lower scale in the entire sample. Men resulted significantly more conservative than women (Chia et al., 1994); this is simply interpretable as the residual of the male/female Italian cultural stereotype.

To summarize, our results (**Table 1**) suggested that (a) fathers and mothers have a similar perception of family functioning, (b) share common cultural attitudes, beliefs and values toward the family, and (c) fathers have a better self-perception of parental role, in terms of investment, sense of competence, role balance, and satisfaction.

Mainly due to differences reported in self-perception of parenting between mothers and fathers, the Pearson product-moment correlations between parenting and cultural issues were carried out separately for mothers and fathers (**Table 2**). Only correlations with medium (>0.30) or large (>0.50) effect size were interpreted.

**Table 2:** Correlations.

		*SPPR* Role Balance	*SPPR* Satisfaction	FAM-III Tot
FAM-III Tot	Mothers	–0.36^∗^	–0.44^∗^	
	Fathers	–0.41^∗^	–0.55^∗^	
				
AFS Tot	Mothers	0.12	0.16	–0.20
	Fathers	0.18	0.22	
Support	Mothers	0.14	0.20	–0.18
	Fathers	0.24	0.25	
Interconnectedness	Mothers	0.20	0.29	–0.30^∗^
	Fathers	0.30^∗^	0.42^∗^	0.27^∗^
Honor	Mothers	–0.12	0.20	
	Fathers			
Subjugation	Mothers		0.10	
	Fathers	–0.10	–0.15	0.17
CVS				
Family solidarity	Mothers	0.37^∗^	0.35^∗^	–0.43^∗^
	Fathers	0.30^∗^	0.51^∗∗^	–0.38^∗^
Executive male attitude	Mothers	–0.28	–0.36^∗^	0.45^∗^
	Fathers	–0.41^∗^	–0.42^∗^	0.41^∗^
Conscience	Mothers	0.15	0.12	–0.12
	Fathers		0.13	–0.13
Equality of sexes	Mothers	0.24	0.37^∗^	–0.42^∗^
	Fathers	0.46^∗^	0.63^∗∗^	–0.48^∗^
Independence	Mothers	0.16		–12
	Fathers	0.17	0.16	
Temporal farsightedness	Mothers	0.14		–0.10
	Fathers	0.23	0.21	
Spousal Employment	Mothers		–0.19	0.24
	Fathers	–0.22	–0.33^∗^	0.27

*Effect size: small between 0.10 and 0.30; ^∗^medium between 0.31 and 0.50; ^∗∗^large higher than 0.50*.

Correlations between SSPR and FAM-III showed that the attributions of parenting satisfaction and role balance in mothers and fathers were related with a positive perception of families. Parents resulted satisfied about their role and are able to balance between being a parent and other kind of role satisfaction; they showed a positive overall perception of being able to meet goals, and to performance a variety of maintenance, developmental, and crisis tasks in the family.

In respect with AFS, attribution of interconnectedness was related with a positive general perception of their family, with role balance and satisfaction in parenting just for fathers: as far as they feel family members must keep in close emotional and physical contact with other family members, they are more able to balance between their role as parents and other roles. Instead, for mothers, interconnectedness was significantly related with a positive perception of family.

Looking at CVS, attributions in Family Solidarity were significantly related to role satisfaction, role balance, and overall family perception for both mothers and fathers. Executive Male Attitude was negatively related with all role and family variables for both parents, except the role satisfaction for mothers; moreover, stronger was the stereotype more negative result family perception for both parents. Attributions of Equality were related with all role and family variables for both parents, except the role satisfaction for mothers. Lastly, just for fathers emerged an inverse correlation between spousal employment and parenting satisfaction, meaning that fathers with the opinion that each spouse should decide about his/her own job, and not about his/her spouse’s job, reported higher levels of satisfaction in parenting. No other values were significantly correlated with role and family dimensions.

In general, although mothers and fathers showed a similar path of correlations, Cohen’s effect sizes were higher in fathers, suggesting stronger relationships between parenting, family functioning, and cultural issues. In both parents, greater level of satisfaction in parenting and better ability in role balancing correlated with a more positive family adjustment. On the other hand, no significant correlations were found between family adjustment and self-perception of competence and investment in parenting.

## Conclusion

Cross-cultural studies have often underscored how parenting self-perception and family perception may be related to parent’s own cultural heritage. However, few empirical studies were carried out to assess specific information due to cultural differences attributed to countries. Literature seems limited to large distinction between Eastern and Western Countries, or between Latinos, Asian and Western/US countries. On the other hand, Triandis (2002) posited how cultural differences would overcome large cultural differences and also that different meaning had to be attributed to the measures of these differences.

Both levels of society, large community and close family group, give their own values to kids during their individual development, that gradually structure their system of belief and behavior, based on these teachings. At the community level, the institutions of which the child is part during his development (school, friends, sports teams, and cultural, etc.) pass on their values, influencing choices and believes about priorities and important aspects of life. The same process of values transmission occurs at the level of close social environment, the family, which, in everyday life, informs the child and then adolescent its own traditions and beliefs, educating him in accordance with these principles. The set of values and beliefs so transmitted at a social level, influence the importance the individual assigns to different elements of his life, specifically to the family, and, consequently, the way in which individuals manage family structure (such as married life, child care, the division of roles within the family, relationships and time spent with their children and other family members). The family structure, with its relationships between members, constitutes the primary environment in which the child grows becoming adolescent, and where he takes the instruments for his psychosocial adaptation. Also for these reasons, family group is therefore an important element of mediation between the cultural values transmitted at the level of extended community, and what actually the child and then the adolescent assimilate within their own cultural knowledge.

Drawing from conceptual links, we tested the hypothesis that familism and culture values about family contributes to parental role and family perception in parents. Parents and families represent a basic and indispensable way in which culture is transmitted to offsprings and this acquires an important role in a specific stage of development as is adolescence. This study was descriptive and exploratory. The aim was simply to have a picture of a limited number of parenting and family measures, to describe them in a sample of Italian parents and to relate them to some cultural measures. All tools were used for the first time in a sample of Italian parents, with exception of FAM-III. Mothers and fathers showed a balanced profile of family functioning. They seem to have the same perspective regarding the quality of integration between family roles, and the willingness of family members to assume the assigned roles. This result was coherent with the literature on family relationships that reports small differences between fathers and mothers in perceiving family functioning (Svetina et al., 2011). It was also coherent with previous results in a large Italian sample of parents (Laghezza et al., 2014). However, it could be also due to the possibility to complete the questionnaires at home, where parents are free to discuss and compare their answers. Shifting to children’s gender, in line with previous studies, this study confirmed that parents’ perception of family functioning did not result to be affected by their children’s gender, at least as it is measured by FAM-III (Tiffin et al., 2007).

Descriptively, results of all the other variables used in this study allow having the first “normative picture” of parenting and cultural variables in a non-clinical sample of Italian adolescents’ parenting. However, the most interesting results concern some identified relationship between parenting and family perception and cultural variable.

Generally, parents who maintain benevolent relationships, common goals with others, social appropriateness, sociability and cooperation, are more satisfied of the role. They are more able to balance roles and show a more positive perception of family, in particular concerning overall positive perception of the family. About familism, only some dimensions, and in particular interconnectedness, influenced parenting and family perceptions. Also regarding family cultural values, only some of them influenced parenting and family perception. In respect with associations between self-perception of parental role and cultural beliefs, the first evidence was that, in both parents, the concepts measured by satisfaction in parenting and role balance subscales seem much more linked to cultural issues than the self-perception about parents’ competence or involvement with the child. In specific, for both parents, higher levels of satisfaction were associated to higher values in family solidarity, equality among sexes and in decision takers. Looking at role balance subscale in mothers, higher scores in balancing the possible different role of life were associated only to higher levels of family solidarity. On the contrary, in fathers it positively correlated with higher scores in family solidarity, equality of the sexes, and equality in decision takers.

These results suggest the importance to study specific cultural variables besides overall general ones to better understand the complex context in which a family is framed. However, the most striking results were that attribution of these cultural variables were stronger related in fathers than mothers. A possible explanation is that fathers, in this specific phase of life, may better contribute to adolescents’ individualization and separation from the family (Bögels and Phares, 2008).

The deepening knowledge of these cultural differences will promote greater awareness among operators, regardless of what the standard of management in families from different cultures, providing them with means of assessment and intervention for family systems, which is sensitive to the cultural background of family under consideration. In particular, it suggests how much important it is to make connection with fathers’ (mostly positive) views as a contribution to family and parenting functioning. Such knowledge will also allow the assessment of the significance of the impact that certain family structures may have on adolescents, according to their own culture, and be a help in understanding the adaptation difficulties of boys from immigrant families.

This paper has many limitations. First, data are a merely description of a group of Italian adolescents’ parents. No more complex statistical analyses were carried out to study relationships about the examined variables. Second, data were collected in intact and non-clinical medium socio-economic status parents of North and Central Italy. More data need to be collected for instance in southern Italy as well as considering divorced or dysfunctional families. Third, no comparison was done with results obtained in previous studies with the same tools in Italy neither in other Eastern or Western countries. Fourth, the paper took in consideration only parents of adolescents. Future studies need to consider parents of younger child in order to improve the generalizability of current results. In addition, another limitation arises from the use of self-report measures, that introduces issues of potential reporter-bias and shared method variance. Additional assessment modalities (e.g., structured interviews) together with self-report measures, can contribute to a more objective and accurate understanding of the phenomena. However, the results for the first time introduced empirical cultural data in connection with parenting and family perception in Italy, showing some important influences, which need to be taken into account in future study.

## Conflict of Interest Statement

The authors declare that the research was conducted in the absence of any commercial or financial relationships that could be construed as a potential conflict of interest.

## Footnotes

1 Demographic information tapping personal and employment related questions was collected with questionnaires. A question referring to participant and his/her child’s previous (within the past 6 months) and/or present psychiatric assessment and/or treatment was asked to both mothers and fathers.

## References

[B1] Abdou K. Parker D. J. Brooks B. Kalthoff N. Lebel T. (2010). The diurnal cycle of lower boundary-layer wind in the West African monsoon. *Q. J. R. Meteorol. Soc.* 136 66–76. 10.1002/qj.536

[B2] Abu-Rayya H. Yang B. (2012). Unhealthy family functioning as a psychological context underlying Australian children’s emotional and behavioural problems. *Int. J. Mental Health.* 8:1.

[B3] Amato P. R. (2005). The impact of family formation change on the cognitive, social, and emotional well-being of the next generation. *Fuct. Child* 15 75–96. 10.1353/foc.2005.0012 16158731

[B4] American Psychological Association (2010). *Ethical Principles of Psychologists and Code of Conduct.* Washington, DC: American Psychological Association.

[B5] Angley M. Divney A. Magriples U. Kershaw T. (2015). Social support, family functioning and parenting competence in adolescent parents. *Matern. Child Health J.* 19 67–73. 10.1007/s10995-014-1496-x 24833286 PMC4233010

[B6] Armstrong M. I. Birnie-Lefcovitch S. Ungar M. T. (2005). Pathways between social support, family well-being, quality of parenting, and child resilience: what we know. *J. Child Fam. Stud.* 14 269–281. 10.1007/s10826-005-5054-4

[B7] Bandura A. (1997). “Self-efficacy and health behavior,” in *Cambridge Handbook of Psychology, Health and Medicine*, eds Baum A. NewmanNewman S. Wienman J. West R. McManus C. (Cambridge: Cambridge University Press), 160–162.

[B8] Barnes G. M. Hoffman J. H. Welte J. H. Farrell M. P. Dintcheff B. A. (2006). Effects of parental monitoring and peer deviance on substance use and delinquency. *J. Marriage Fam.* 68 1084–1104. 10.1111/j.1741-3737.2006.00315.x

[B9] Bögels S. M. Phares V. (2008). Fathers’ role in the etiology, prevention and treatment of child anxiety: a review and new model. *Clin. Psychol. Rev. Psychol. Rev.* 28 539–558. 10.1016/j.cpr.2007.07.011 17854963

[B10] Bornstein M. H. (2002). “Parenting infants,” in *Handbook of Parenting, Children and Parenting* Vol. 1 2nd Edn, ed. Bornstein M. H. (Mahwah, NJ: Lawrence Erlbaum Associates), 3–43.

[B11] Bornstein M. H. Cote L. R. Venuti P. (2001). Parenting beliefs and behaviors in northern and southern groups of Italian mothers of young infants. *J. Fam. Psychol. Fam. Psychol.* 15 663–675. 10.1037/0893-3200.15.4.663 11770473

[B12] Bornstein M. H. Hahn C. Haynes O. M. (2011a). Maternal personality, parenting cognitions and parenting practices. *Dev. Psychol.* 47 658–675. 10.1037/a0023181 21443335 PMC3174106

[B13] Bornstein M. H. Putnick D. L. Lansford J. E. (2011b). Parenting attributions and attitudes in cross-cultural perspective. *Parent. Sci. Pract.* 11 214–237. 10.1080/15295192.2011.585568 21927591 PMC3173779

[B14] Bornstein M. H. Hendricks C. Hahn C. S. Haynes O. M. Painter K. M. Tamis-LeMonda C. S. (2003). Contributors to self-perceived competence, satisfaction, investment, and role balance in maternal parenting: a multivariate ecological analysis. *Parent. Sci Pract.* 3 285–326. 10.1207/s15327922par0304_2

[B15] Bradshaw J. Richardson D. (2009). An index of child well-being in Europe. *Child Indic Res.* 2 319–351. 10.1007/s12187-009-9037-7

[B16] Brown S. L. (2004). Family structure and child well-being: the significance of parental cohabitation. *J. Marriage Fam.* 66 351–367. 10.1111/j.1741-3737.2004.00025.x

[B17] Campos B. Schetter C. D. Abdou C. M. Hobel C. J. Glynn L. M. Sandman C. A. (2008). Familialism, social support, and stress: positive implications for pregnant Latinas. *Cultur. Divers Ethnic Minor. Psychol.* 14 155–162. 10.1037/1099-9809.14.2.155 18426288 PMC2859297

[B18] Campos R. (2008). Considerations for studying father involvement in early childhood among Latino families. *Hisp. J. Behav. Sci.* 30 133–160. 10.1177/0739986308316658

[B19] Cardoso J. B. Thompson S. (2010). Common themes of resilience among Latino immigrant families: a systematic review of the literature. *Fam. Soc.* 91 257–265. 10.1606/1044-3894.4003

[B20] Chia R. C. Wuensch K. L. Childers J. Chuang C. Cheng B. Cesar-Romero J. (1994). A comparison of family values among Chinese, Mexican, and American college students. *J. Soc. Behav. Personal.* 9 249–258.

[B21] Cohen J. (1988). *Statistical Power Analysis for the Behavioral Sciences*, 2nd Edn. Hillsdale, NJ: Lawrence Erlbaum Associates, Publishers.

[B22] Coltrane S. Parke R. D. Adams M. (2004). Complexity of father involvement in low-income Mexican-American Families. *Fam. Relat.* 53 179–189. 10.1111/j.0022-2445.2004.00008.x

[B23] Delvecchio E. Di Riso D. Chessa D. Salcuni S. Mazzeschi C. Laghezza L. (2014). Expressed emotion, parental stress, and family dysfunction among parents of nonclinical Italian children. *J. Child Fam. Stud.* 23 989–999. 10.1007/s10826-013-9754-x

[B24] Department of Families Housing Community Services and Indigenous Affairs (2010). *Better Start-Early Intervention for Children with Disability Initiative.* Available at: https://www.dss.gov.au/

[B25] Dishion T. J. Bullock B. M. Kiesner J. (2008). “Vicissitudes of parenting adolescents: daily variations in parental monitoring and the early emergence of drug use,” in *What Can Parents Do? New Insights Into the Role of Parents in Adolescent Problem Behavior*, eds Kerr M. Stattin H. Engels R. C. M. E. (Chichester: Wiley), 113–133.

[B26] Erkut S. (2010). Developing multiple language versions of instruments for intercultural research. *Child Dev. Perspect.* 4 19–24. 10.1111/j.1750-8606.2009.00111.x 21423824 PMC3060794

[B27] Ferrari A. M. (2002). The impact of culture upon child rearing practices and definitions of maltreatment. *Child Abuse Negl.* 26 793–813. 10.1016/S0145-2134(02)00345-9 12363332

[B28] Ferro M. A. Boyle M. H. (2014). The impact of chronic physical illness, maternal depressive symptoms, family functioning, and self-esteem on symptoms of anxiety and depression in children. *J. Abnorm. Child Psychol.* 43 177–187. 10.1007/s10802-014-9893-6 24938212

[B29] Germán M. Gonzales N. A. Dumka L. (2009). Familism values as a protective factor for Mexican-origin adolescents exposed to deviant peers. *J. Early Adolesc.* 29 16–42. 10.1177/0272431608324475 21776180 PMC3138713

[B30] Hollingshead A. A. (1975). *Four-Factor Index of Social Status.* New Haven, CT: Yale University.

[B31] Hsu H.-C. Lavelli M. (2005). Perceived and observed parenting behavior in American and Italian first-time mothers across the first 3 months. *Infant Behav. Dev.* 28 503–518. 10.1016/j.infbeh.2005.09.001

[B32] Kumpfer K. L. Alvarado R. Smith P. Bellamy N. (2002). Cultural sensitivity and adaptation in family-based prevention interventions. *Prev. Sci.* 3 241–246. 10.1023/A:1019902902119 12387558

[B33] Laghezza L. Delvecchio E. Pazzagli C. Mazzeschi C. (2014). The family assessment measure III (FAM III) and parental stress in an Italian sample. *Boll. Psicol. Appl.* 4 17–28.

[B34] Letourneau N. L. Tramonte L. Willms J. D. (2013). Maternal depression, family functioning and children’s longitudinal development. *J. Pediatr. Nurs.* 28 223–234. 10.1016/j.pedn.2012.07.014 22940454

[B35] Lugo Steidel A. G. Contreras J. M. (2003). A new familism scale for use with latino populations. *Hisp. J. Behav. Sci.* 25 312–330. 10.1177/0739986303256912

[B36] MacPhee D. Benson J. B. Bullock D. (1986). Influences on maternal self-perceptions. *Infant Behav. Dev.* 9:236 10.1016/S0163-6383(86)80239-9

[B37] Marsiglio W. Amato P. Day R. D. Lamb M. E. (2000). Scholarship on fatherhood in the 1990s and beyond. *J. Marriage Fam.* 62 1173–1191. 10.1111/j.1741-3737.2000.01173.x

[B38] McBride B. A. Dyer W. J. Liu Y. Brown G. L. Hong S. (2009). The differential impact of early father and mother involvement on later student achievement. *J. Educ. Psychol.* 101 498–508. 10.1037/a0014238 25414521 PMC4235963

[B39] Nelson S. K. Kushlev K. Lyubomirsky S. (2014). The pains and pleasures of parenting: when, why, and how is parenthood associated with more or less well-being? *Psychol. Bull.* 140 846–895. 10.1037/a0035444 24491021

[B40] Newland L. A. (2015). Family well-being, parenting, and child well-being: pathways to healthy adjustment. *Clin. Psychol.* 19 3–14. 10.1111/cp.12059

[B41] Peña E. D. (2007). Lost in translation: methodological considerations in cross-cultural research. *Child Dev.* 78 1255–1264. 10.1111/j.1467-8624.2007.01064.x 17650137

[B42] Perry-Jenkins M. Repetti R. L. Crouter A. C. (2000). Work and family in the 1990s. *J. Marriage Fam.* 62 981–998. 10.1111/j.1741-3737.2000.00981.x

[B43] Pleck J. H. (2007). Why could father involvement benefit children? Theoretical perspectives. *Appl. Dev. Sci.* 11 196–202. 10.1080/10888690701762068

[B44] Pleck J. H. Hofferth S. (2008). Mother involvement as an influence on father involvement with early adolescents. *Fathering* 6 267–286. 10.3149/fth.0603.267 21776195 PMC3138554

[B45] Romero A. J. Ruiz M. (2007). Does familism lead to increased parental monitoring? Protective factors for coping with risky behaviors. *J. Child Fam. Stud.* 16 143–154. 10.1007/s10826-006-9074-5

[B46] Roosa M. Morgan-Lopez A. Cree W. Specter M. (2005). Family and child characteristics linking neighborhood context and child externalizing behavior. *J. Marriage Fam.* 67 514–529. 10.1111/j.0022-2445.2005.00132.x

[B47] Sabogal F. Marin G. Otero-Sabogal R. Marin B. Perez-Stable E. (1987). Hispanic familism and acculturation: what changes and what doesn’t? *Hisp. J. Behav. Sci.* 9 397–412. 10.1177/07399863870094003

[B48] Scaramella L. V. Conger R. D. Simons R. L. (1999). Parental protective influences and gender-specific increases in adolescent internalising and externalising problems. *J. Res. Adolesc.* 9 111–141. 10.1207/s15327795jra0902_1

[B49] Schwartz S. J. (2007). The structure of identity consolidation: multiple correlated constructs or one superordinate construct? *Identity Int. J. Ther. Res.* 7 27–49. 10.1080/15283480701319583

[B50] Schwartz S. J. Weisskirch R. S. Hurley E. A. Zamboanga B. L. Park I. J. K. Kim S. Y. (2010). Communalism, familism, and filial piety: are they birds of a collectivist feather? *Cultur. Divers Ethnic Minor Psychol.* 16 548–560. 10.1037/a0021370 21058818 PMC7869140

[B51] Seybold J. Fritz J. MacPhee D. (1991). Relation of social support to the self-perceptions of mothers with delayed children. *J. Commun. Psychol.* 19 29–36. 10.1002/1520-6629(199101)19:1<29::AID-JCOP2290190104>3.0.CO;2-4

[B52] Skinner H. Steinhauer P. Santa-Barbara J. (1983). The family assessment measure. *Can. J. Commun. Mental Health* 2 91–103. 10.7870/cjcmh-1983-0018

[B53] Skinner H. Steinhauer P. Sitarenios G. (2000). Family assessment measure (FAM) and process model of family functioning. *J. Fam. Ther.* 22 190–210. 10.1111/1467-6427.00146

[B54] Smetana J. G. Crean H. F. Daddis C. (2002). Family processes and problem behaviours in middle-class African American adolescents. *J. Res. Adolesc.* 12 275–304. 10.1111/1532-7795.00034

[B55] Statham J. Chase E. (2010). *Childhood Wellbeing: A Brief Overview.* London: Childhood Wellbeing Research Centre.

[B56] Svetina M. Zebret E. Bajec B. (2011). Perception of family functioning: parental vs. non-parental perspective. *Suvr. Psihol.* 14 5–15.

[B57] Taylor Z. E. Larsen-Rife D. Conger R. D. Widaman K. F. (2012). Familism, interparental conflict, and parenting in mexican-origin families: a cultural-contextual framework. *J. Marriage Fam.* 74 312–327. 10.1111/j.1741-3737.2012.00958.x 22736810 PMC3378250

[B58] Tiffin P. A. Pearce M. S. Kaplan C. Fundudis T. Parker L. (2007). Recollections of parental style and perceptions of current family functioning at age 50. *J. Fam. Ther.* 29 169–182. 10.1111/j.1467-6427.2007.00379.x

[B59] Triandis H. (2002). Individualism-collectivism and personality. *J. Pers.* 69 907–924. 10.1111/1467-6494.696169 11767823

[B60] Van de Vijver F. Hambleton R. K. (1996). Translating tests: some practical guidelines. *Euro. Psychol.* 1 89–99. 10.1027/1016-9040.1.2.89

[B61] Vieno A. Nation M. Pastore M. Santinello M. (2009). Parenting and antisocial behavior: a model of the relationship between adolescent self-disclosure, parental closeness, parental control, and adolescent antisocial behavior. *Dev. Psychol.* *Psychol.* 45 1509–1519. 10.1037/a0016929 19899910

[B62] Waldman-Levi A. Finzi-Dottan R. Weintraub N. (2015). Attachment security and parental perception of competency among abused women in the shadow of PTSD and childhood exposure to domestic violence. *J. Child Fam. Stud.* 24 57–65. 10.1007/s10826-013-9813-3

[B63] Winek J. L. (2010). *Systemic Family Therapy: From Theory to Practice.* Los Angeles, CA: Sage.

